# Chemistry

**Published:** 1899-11

**Authors:** 


Chemistry. General, Medical and Pharmaceutical, including the Chemistry of the United States Pharmacopœia. By John Attfield, F. R. S. New (16th) edition. In one royal 12mo. volume of 784 pages, with 88 illustrations. Cloth, $2.50, net. Lea Brothers & Co., Philadelphia and New York.

It is saying a great deal for a book when it can be announced that the sixteenth edition is ready to market. This work, for so long the standard in all countries, has been thoroughly revised and brought up to date. It accords with the last edition of the United States Pharmacopœia, and needs no introduction. For thirty-one years *Attfield's Chemistry* has been the trustworthy guide of students everywhere. A warm welcome awaits this edition. The price, notwithstanding the excellency of the work has been enhanced, has been reduced to meet the popular demand. B.

